# Perspective-Taking and Reactions Toward Poor Performers in Groups: A Scoping Review and Discussion

**DOI:** 10.3390/bs15050612

**Published:** 2025-05-01

**Authors:** Emma Halfmann, J. Lukas Thürmer

**Affiliations:** 1 Department of Psychology, Paris Lodron University Salzburg, 5020 Salzburg, Austria; lukas.thuermer@plus.ac.at; 2 Department of Psychology, Justus-Liebig University of Giessen, 35390 Giessen, Germany; 3 Salzburg Center of European Union Studies, Paris Lodron University Salzburg, 5020 Salzburg, Austria; 4 Economic Psychology Professorship, Private University Seeburg Castle, 5201 Seekirchen am Wallersee, Austria

**Keywords:** perspective-taking, attribution, poor performers, pro-group intent

## Abstract

Perspective-taking, the ability to adopt another person’s viewpoint, has been found to enhance group performance by fostering cooperation and coordination. However, if members threaten the attainment of group goals (i.e., *poor performers*), the intensity of perspective-taking is not sufficient to explain group members’ reactions to the poor performer (e.g., willingness to punish), since the findings are not unequivocally positive. It is key to consider the inferences resulting from perspective-taking efforts (attributions). These inferences, as attributions of the cause of the poor performance and the pro-group intent, are key determinants of group responses to poor performers. The goal of this scoping review is to examine the role of perspective-taking and attributions of the cause of poor performance in reactions toward poor performers in groups. Following the PRISMA guidelines for scoping reviews, we performed a literature search in three databases (APA PsycInfo, PubPsych, and Web of Science) that yielded ten articles that matched our eligibility criteria. A narrative synthesis was employed to summarize the main findings across the included literature. This review highlights the need for integrating views on perspective-taking and attribution processes in group contexts to better understand how groups can effectively navigate challenges posed by diverging performance.

## 1. Introduction

Perspective-taking is a core socio-cognitive skill that enables individuals to infer and understand others’ mental states, intentions, emotions, and viewpoints (Davis, 1983). It plays a central role in social functioning and coordination, cooperation, and conflict resolution (Galinsky et al., 2005). The social world is inherently complex, as humans commonly cooperate with a host of different people. They often engage in diverse groups composed of individuals with varying abilities, motivations, and opinions. These differences can enrich group interactions but also pose significant challenges. To yield the benefits of such social bonds, we need to successfully manage our different perspectives, opinions, and abilities. Understanding others’ thoughts and feelings is crucial for navigating complex social interactions effectively, as it enables individuals to make sense of behaviors and opinions that differ from their own (cf. Galinsky et al., 2005; Perner, 1988). Without this ability, all interactions would be fraught with misunderstandings and conflict. But to function effectively, groups must foster cooperation and coordinate members’ behavior to perform tasks and achieve collective goals. This is further complicated by the inherent social dilemma that group work poses, where all members benefit from group success, regardless of the quality and degree of individual contributions to it (cf. Kerr, 1983; Levine & Moreland, 1994). Equity norms expect everyone to contribute equally if they benefit equally from a group task. However, these can be violated when performance inequalities occur, as in the example of poor performance of a team member. Following the rationale of the *bad apple effect* (Felps et al., 2006), a single divergent team member suffices to disrupt team effectiveness and can increase the potential for conflict. On other occasions, groups have been observed to respond leniently toward poor performers by compensating for or training them (Jackson & LePine, 2003; Kerr, 1983).

But how do groups determine their response to poor performers? Some research suggests that perspective-taking helps overcome obstacles that arise when working together. Building on the fundamental capacity of identifying the mental states of others (*Theory of Mind*; cf. Wellman, 2014), perspective-taking is the active use of this ability to take another’s point of view into account. Many studies manipulating the intensity of perspective-taking have observed positive effects of perspective-taking (see Caruso et al., 2006a). Yet, the underlying processes of perspective-taking in groups are not fully understood, as evident in the mixed findings of its effects. Specifically, other studies have found increased negative reactions towards team members (cf. Caruso et al., 2006a, 2006b). Thus, the observers’ efforts to understand the reasons for a target’s poor performance have mixed effects on group reaction.

Other research suggests that group reactions to poor performance should be determined not only by the intensity of perspective-taking efforts but instead by the outcome of such a process. Inferring reasons for actions is an act of perspective-taking (Malle, 2011). Attribution theory assumes that two factors can cause poor performance: low effort and low ability (e.g., Weiner, 1972, 1993). When group members engage in perspective-taking and draw inferences about the cause of a poor performance, the poor performer is judged more harshly when the cause is attributed to low effort. Effort is viewed as more controllable than ability, and the poor performer is therefore perceived as more responsible, which in turn leads to more negative responses from the team members.

Combining both views, the outcome of increased perspective-taking should crucially depend on the inferences reached about the target. For example, if a group is working together on a goal and realizes that one person is not adequately contributing to the group goal, putting yourself in that person’s shoes could increase negative reactions to that person if the group attributes selfish reasons to the individuals’ poor performance (Thürmer & Kunze, 2023).

In the current scoping review paper (cf. Arksey & O’Malley, 2005), we aim to systematically map and describe how perspective-taking and attribution processes shape group reactions to a poor performer. Additionally, we aim to evaluate the impact of attribution processes on peer responses. The scope of our review is, therefore, to compile the evidence from research on perspective-taking and attribution for the cause of performance to comprehensively evaluate the socio-emotional, cognitive, and behavioral reactions of team members towards a poor-performing teammate. As a result, we are able to pinpoint research gaps that need to be filled to advance the knowledge in this domain. We synthesize research in the fields of perspective-taking in social interactions, attribution of intent, and task group responses to diverging behaviors (i.e., *poor performance*). These streams of work concur that attributing the intentions (goals) of group members via perspective-taking plays a key role in group dynamics and offers an explanation of the negative results of perspective-taking instructions in group contexts (cf. Caruso et al., 2006a). Specifically, we highlight the central role that a performer’s attributed intent to help the group (*pro-group intent*) plays in shaping how the group coordinates, cooperates, and manages challenges arising from individual behavior that may deviate from group norms or goals (Thürmer & Kunze, 2023). The M-PGI focuses not only on the observable characteristics (effort/ability) but also on the subjective goal-setting process (desirability/feasibility). Attributing a poor performance to low ability and low feasibility should result in a less negative reaction than attributing it to low effort and low desirability. Attributing poor performance to the common precursors (effort or ability) influences the perceived pro-group intent and shapes the following reactions aimed at ensuring collective goal attainment. Taken together, perspective-taking should lead to highly nuanced attributions of causes for poor performance and—following the M-PGI—consequently, the ascription of high or low pro-group intent. In turn, the degree of attributed pro-group intent shapes the group’s reactions. We discuss the evidence of our scoping review and shed light on how perspective-taking, attribution processes, and consequent responses shape group dynamics in reaction to poor performance.

## 2. Poor Performers in Task Groups

Following the rationale of the *collective action control model* (Thürmer et al., 2024), humans have two distinctive “superpowers”: the collective pursuit of goals and an unparalleled capacity for cooperation among known life forms. Self-control and self-regulation—executive functions enabling goal attainment—play a critical role in adhering to societal norms, engaging in costly cooperation, and interacting effectively with others. These abilities represent a unified capacity: achieving shared goals cooperatively. In this sense, groups regulate the behavior of their members, a process now commonly referred to as *collective action control* (Thürmer et al., 2024).

Humans interact in diverse groups, including members with different abilities, task-relevant knowledge, skills, motivations, perspectives, and opinions (Reinert et al., 2024; Van Knippenberg & Schippers, 2007). This diversity allows groups to draw on a larger pool of resources and can enable more creative and innovative group performance (e.g., Bantel & Jackson, 1989). Yet, team diversity poses a challenge (Hoever et al., 2012). Group members must cooperate and thus rely on their members’ contributions to achieve collective goals. Group work inherently poses a social dilemma of contributing to joint success or selfishly withholding contributions while participating in group success (cf. Kerr, 1983; Levine & Moreland, 1994). Accordingly, while most team members contribute their fair share, others may perform poorly (e.g., because their personal goals diverge from team goals). To illustrate this, consider a sales team in an industrial production company charged with developing a presentation for a sales pitch for a client at the end of the week. The team members all receive an equal bonus for completing the sale. However, one team member contributes substantially less effort and work time to preparing the pitch than the others, neglecting core tasks such as preparing the slides carefully and rehearsing the presentation. In situations like this, groups are typically sensitive to individuals exerting less effort as this threatens group goal attainment and productivity (cf. Karau & Wilhau, 2020). As a result, well-performing team members may reduce their effort when a poor performer is present (*sucker effect*; Kerr, 1983). In fact, a single poor performer can significantly decrease team and organizational outcomes, such that performance and social interaction quality suffer (*bad apple effect*; Felps et al., 2006).

Poor performance can elicit negative socio-emotional responses in the group, and behavioral reactions range from offering support and compensation to excluding or punishing the performer (Jackson & LePine, 2003; LePine & Van Dyne, 2001). These reactions likely have downstream consequences and are important determinants of the group’s performance success and goal attainment. How groups perceive and react to deviant performances and behavior in teams is thus an important aspect of collective action control. While individual and social properties (e.g., social identity; see Hütter & Diehl, 2011) may influence team reactions to poor performance, we are interested in the effect of perspective-taking and the content of the consequent attributions, since considering the perspective of another person typically has beneficial effects on cooperation and coordination in groups and interactions. Putting yourself in the position of the poor performer and trying to understand their thoughts and emotions may, therefore, help to overcome the challenges posed by poor performance. But how exactly can groups infer the mental states of their members? The basis for this process may be the ability to consider the world from another person’s point of view (Theory of Mind).

## 3. Perspective-Taking in Task Groups

Theory of Mind (TOM) refers to a set of interrelated skills that enable people to understand the minds of others (Wellman, 2014). Perspective-taking is one process related to TOM that encompasses the capacity to consider the world from another person’s viewpoint and infer the content of their perceptions, thoughts, and feelings (Davis, 1983). Via perspective-taking, individuals can anticipate the behavior and reactions of others, a cornerstone of social competence (Davis, 1983). While understanding others is the goal of perspective-taking, it is typically operationalized in terms of intensity, rather than through the concrete inferences it may produce. Early accounts of perspective-taking entailed a strong emotional component, demonstrating its importance for empathy (Galinsky & Moskowitz, 2000). While empathy and perspective-taking are correlatively and causally interrelated (Batson, 1991), it is important to stress that both are conceptually distinct. Perspective-taking has been argued to be an intellectual process rather than an emotional reaction (Ku et al., 2015). While individuals with high empathy are more accurate in understanding others’ emotions, a high intensity of perspective-taking helps to understand others’ cognitions (Gilin et al., 2013). The mechanisms behind perspective-taking have been described as an active cognitive process with direct cognitive consequences regarding attributional thinking and evaluation (cf. Galinsky et al., 2005). Because perspective-taking allows one to take the perspective of another person, a self–other overlap of mental representations enables individuals to see more of themselves in the other person and more of the other person in themselves. Individuals are prompted to move beyond habitual mental routines and default cognitive processes, allowing them to overcome egocentric perspectives (Epley et al., 2004; Todd et al., 2012; Vescio et al., 2003).

A persistent challenge in groups is individuals’ tendency to prioritize self-interest and withhold cooperation or resources. This often stems from egocentric biases, where individuals overestimate their contributions or feel entitled to a disproportionate share of resources (Caruso et al., 2006a; Epley et al., 2006; Ross & Sicoly, 1979). Consequently, an important theoretical and practical concern is identifying critical mechanisms to decrease social bias, coordinate behavior with others, and create and strengthen social bonds (Galinsky et al., 2005). Perspective-taking has various results that directly affect human interactions (see, for example, Caruso et al., 2006a; Ku et al., 2015). It has fundamental positive effects on the target, resulting in increased liking (Galinsky & Moskowitz, 2000), relational satisfaction (Davis & Oathout, 1987), and psychological closeness (Cialdini et al., 1997; Davis et al., 1996). It promotes prosocial behavior, including generosity and helping (Batson, 1991; Batson et al., 1991; Dovidio et al., 1990; Gino & Galinsky, 2012; Shih et al., 2009) and facilitates behavioral mimicry (Galinsky et al., 2005; Laurent & Myers, 2011), which plays a key role in fostering pleasant social interactions and smoother coordination (Chartrand & Bargh, 1999; Galinsky et al., 2005). Perspective-taking decreases stereotyping and prejudice (Batson et al., 1997; Galinsky & Ku, 2004; Galinsky & Moskowitz, 2000; Vescio et al., 2003), improving outgroup evaluations (Galinsky & Moskowitz, 2000), which is especially valuable in diverse team settings. Perspective-taking facilitates cooperative interactions (Johnson, 1975), shaping problem-solving and decision-making abilities and the performance quality of teams. Without perspective-taking, interactions can result in conflict, disagreement, or suboptimal decisions (Caruso et al., 2006a; Todd & Galinsky, 2014).

Perspective-taking can reduce egocentric fairness judgments and, in group contexts, diminish self-serving behaviors, such as taking fewer shared resources (Drolet et al., 1998; Epley et al., 2006). Perspective-takers share more information with team members, resulting in cooperative behavior and effective communication (Falk & Johnson, 1977; Galinsky et al., 2014). Even in competitive conflict resolution tasks like negotiations, the active consideration of the viewpoint of the opponent bears a powerful advantage. Perspective-taking is a precursor to the exchange and processing of interest-related information, as it facilitates inferences about the other party’s potentially divergent interests (e.g., Kemp & Smith, 1994; Neale & Bazerman, 1983). Perspective-taking mitigates the risk of partial impasses and enhances joint outcomes by overcoming selfish motivations (Galinsky et al., 2008; Hoever et al., 2012; Trötschel et al., 2011). Taken together, considering another person’s point of view is an important aspect of regulating team member behavior and facilitating coordination and cooperation in pursuing collective goals.

Despite the extensive evidence for its benefits, perspective-taking can also backfire, resulting in less favorable evaluations, negative emotions, and even exclusion (see Caruso et al., 2006a). Individuals commonly overestimate the extent to which others’ thoughts or behaviors are guided by self-interest (*naive cynicism*; Kruger & Gilovich, 1999; Miller, 1999). Thinking thoroughly about the extent of the group-directed intent of the other person might turn out less favorably, resulting in anger, distrust, and blaming (cf. Schweinle et al., 2002; Sillars et al., 2000). Potentially wrongful attributions may strengthen this impression, and observers may feel that they need to protect themselves and prevent exploitation (cf. *norm of self-interest*; Miller, 1999). This increases the likelihood of negative behavioral reactions such as rejection or punishment, especially in situations with strong competitive incentives (cf. Caruso et al., 2006a). Attributed selfish intentions can also serve as a pretext for behaving more selfishly oneself (cf. Epley et al., 2004). Additionally, the motivation of the group members plays a role. Groups receiving cooperative rather than competitive incentives reacted differently when performances diverged (cf. Caruso et al., 2006b; see also Caruso et al., 2006a for a similar rationale).

## 4. Pro-Group Intent

Perspective-taking can elicit negative reactions toward poor performers under certain circumstances. We argue that when facing poor performers, it is not sufficient to consider the other’s point of view; instead, it is important to consider the content of the attributions of the deviant’s motives and intentions. Put differently, we argue, it is not the sheer intensity of perspective-taking in groups that shapes dynamic response but the specific conclusion and inferences resulting from these considerations. We discuss the Model of Pro-Group Intent (M-PGI) that explicates perspective-taking processes in social interactions, yielding nuanced predictions on how groups respond to poor performers (Thürmer & Kunze, 2023).

To regulate team members’ behavior, groups benefit from considering the perspective of the poorly performing team member and reacting accordingly. When individuals put themselves in another person’s shoes, they actively think about their feelings, thoughts, and intentions. As Malle (1999) argues, intentionality is a key determinant in inferring reasons for a past behavior. This attribution of intent approach has recently been proposed to predict group reaction to poor performers (Thürmer, 2024; Thürmer & Kunze, 2023; Thürmer et al., 2024). Theories on social perceptions and classic attribution research have emerged largely independently of ToM approaches, even though they are conceptually linked. Intentions (or goals; e.g., “I want to attain endstate X”) are important—yet imperfect—predictors of future behavior (Sheeran et al., 2005; Webb & Sheeran, 2006), and individuals are able to infer others’ goals (e.g., Malle, 2011; Malle & Holbrook, 2012).

**Figure 1:**
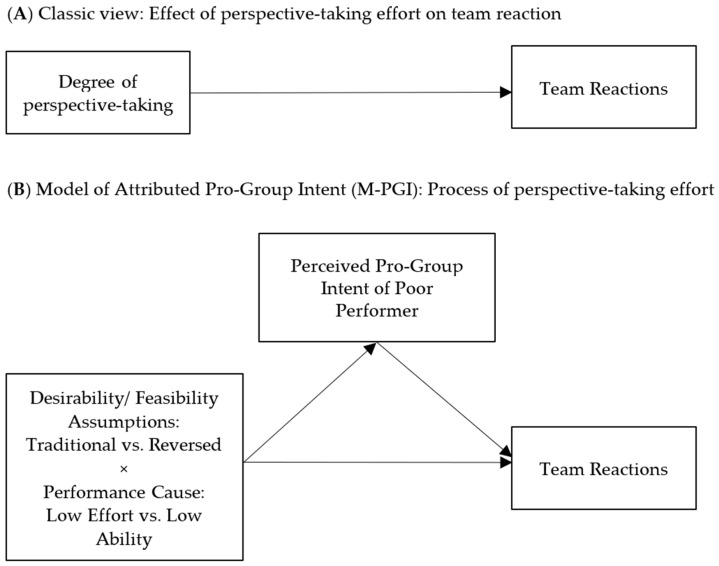
(**A**) Classic view: low effort/low desirability and low ability/low feasibility. (**B**) Reversed assumptions: low effort/low feasibility and low ability/low desirability (adapted from Thürmer, 2024).

The M-PGI focuses on the role of attributed pro-group intent in task groups’ reactions to low-performing members (Thürmer & Kunze, 2023). As groups depend on individual contributions to the group goal (e.g., Kerr, 1983), they should be motivated to infer group members’ intentions. Thus, individuals observe their interaction partners closely and draw causal conclusions to infer the intentionality of their behavior (cf. Malle & Holbrook, 2012). Following classic attribution theory, it is assumed that low effort and low ability can cause poor performance (e.g., Weiner, 1972, 1993). When other group members derive reasons for the poor performance of a member and attribute it to low effort, they are usually judged more harshly than the same performance attributed to low ability (e.g., Carless & Waterworth, 2012; Twardawski et al., 2019; Weiner & Kukla, 1970). This classic view (Figure 1A) ignores perceived intent as an important determinant of group reactions (Figure 1B). Higher-order (or long-term) intentions—such as a performer’s intent to help the group, that is, pro-group intent—are key drivers of behavior and thus may be attributed to actors (Thürmer & Kunze, 2023; Thürmer et al., 2024). Integrating pro-group intent challenges classic attributional models that predominantly focus on cognitive evaluations, such as the controllability of a behavior.

The M-PGI further proposes that traditional assumptions linked to the genesis of effort and ability moderate the effort/ability effect. Individuals typically set goals based on the attractiveness (*desirability*) of a goal and the perceived likelihood of attainment (*feasibility*) (Gollwitzer, 1990; Lewin et al., 1944). Moreover, individual ability can be seen as a malleable or fixed skill (Dweck & Yeager, 2019). Attribution research assumes that effort is perceived to be more controllable than ability, low effort reflects the actor’s belief that the goal is unattainable (low desirability), and low ability reflects the actor’s belief that the lack of skills is unmodifiable (low feasibility; *traditional assumptions*). Following the M-PGI, this is too simplistic. Group members observing poor performers can attribute low effort to a performer’s belief that a goal is unattainable (low feasibility) and low ability to the performer’s lack of desire to acquire the necessary skills (low desirability). Desirability but not feasibility is an important indicator of pro-group intent. Poor performers who ostensibly think that the group goal is not attractive or worth attaining (low desirability) signal a weak pro-group intent. Therefore, the group reactions toward poor performers who show a lack of effort due to low desirability should elicit lower attributions of pro-group intent than low ability due to low feasibility (traditional assumption). When these assumptions are reversed, linking effort to feasibility and ability to desirability, this effect should be eliminated (reversed assumption). The MPGI predicts that the effect of the cause of poor performance (effort/ability) on reactions to poor performers is moderated by the desirability/feasibility assumptions and mediated by attributed pro-group intent.

Taken together, perspective-taking efforts should lead to highly nuanced attributions of causes for poor performance and—following the MPGI—consequently, the ascription of high or low pro-group intent. The degree of pro-group intent matters in group contexts and shapes the group’s reactions. In the following, we present a scoping review of articles analyzing the effects of attribution or perspective-taking on the responses of peers to a low-performing group member.

## 5. Method

### Study Selection Process

The review was not pre-registered but followed the PRISMA reporting guidelines for scoping reviews. To address our research question, we conducted a literature review searching three databases (APA PsycInfo, PubPsych, and Web of Science) for articles published by April 2025. Search terms covered the following topics: (1) poor performance, (2) perspective-taking/Theory of Mind, (3) groups/teams. We used the following list of search terms: (“poor performance” OR “low performance” OR “poor performer” OR “low performer”) AND (“perspective taking” OR “theory of mind” OR “attribution”) AND (group OR team). The keyword searches resulted in 51 entries in Web of Science, 74 entries in APA PsycInfo, and 36 entries in PubPsych. We also conducted an additional search consisting of manual searches across the first five pages from Google Scholar (*n* = 3), and examined relevant cited references of included studies that were of interest (*n* = 3). The articles were uploaded to Covidence (a web-based collaboration software platform that streamlines the production of reviews; Veritas Health Innovation, 2022) and merged, and duplicates (*n* = 62) were removed, resulting in 105 articles that were screened for eligibility.

We only included studies in which the outcome (reactions of other team members to poor performance of a team member) is described and studies in which the independent variables (perspective-taking or attribution) are present and a group/team context is provided. Furthermore, studies not written in English, unpublished doctoral theses, review or theoretical articles, studies addressing the development of Theory of Mind in children (population), those addressing supervisor (and not peer) reactions, and those addressing physical and mental illnesses or disorders (e.g., autism, schizophrenia, brain injuries, etc.) were excluded. Following our eligibility criteria, 85 articles were removed after screening the titles and abstracts. The remaining 20 articles were reviewed as full texts to assess eligibility.

**Figure 2:**
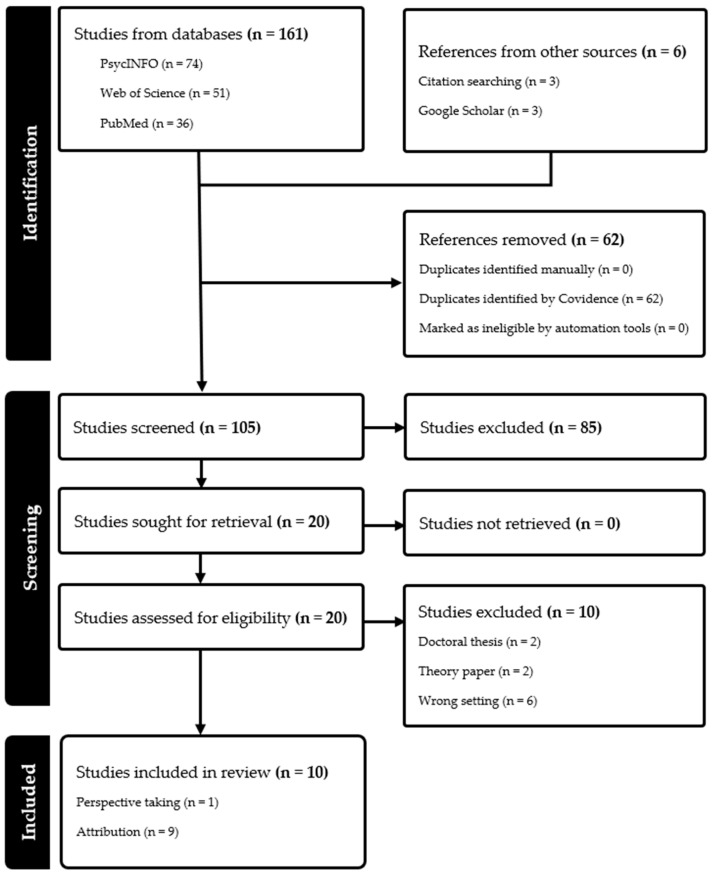
PRISMA flow diagram for our scoping review.

The first author screened and selected the articles, and ten articles were excluded based on the eligibility criteria. The second author reviewed the final selection of ten articles. The study selection process is illustrated in Figure 2. For each article included, we report the following information: publication details, study methods, sample size, design, key measures, and relevant key findings. These details were extracted by the first author (Table S1; Supplementary Material).

## 6. Results

The studies included in this review were not sufficiently homogeneous in aims, measures, and study designs to be suited for a data synthesis (e.g., systematic review or statistical meta-analysis). Instead, a narrative synthesis of the results is presented in the following. We grouped the studies by the type of manipulation (attribution of cause for poor performance vs. perspective-taking) and method (vignette studies vs. interactive group studies). We present study designs and relevant key findings regarding socio-emotional, cognitive, and behavioral reactions of the peer towards the poor-performing team member.

Only one article addresses the effect of a perspective-taking instruction on reactions toward diverging contributions in group tasks. In two experimental studies (Studies 3 and 4), Caruso et al. (2006b) manipulated the focus on the self vs. others and assessed the perceived percentage of individual contributions and reactions towards peers in group tasks. After a perspective-taking instruction to consider others’ contributions, high contributors enjoyed the project less, were less happy with the division of labor, and were less willing to work with the group in the future compared to low contributors. Although the study does not directly manipulate poor performance, it stresses that the result of perspective-taking seems to depend on the individual contribution of the perspective-taker relative to poor performers. Interestingly, when there is no information about the reason for a team failing or no person is identified as responsible, individuals tend to make self-serving attributions, influencing the performance of future collaborations. In an experiment, Reimer (2001) showed that when solving several rounds of group tasks (Tower of Hanoi Problem), individuals primarily attributed the poor performance of the team to their partner. The degree of self vs. other blame was associated with the performance in the subsequent task, indicating that when working in a low-performing team, low performance will not be compensated and will further decline when individuals blame others for the poor performance. These findings diverge from those on the aforementioned positive impact of perspective-taking and emphasize that perspective-taking triggers individual attribution processes that can provoke negative reactions toward low performers.

Following the attribution theories outlined before, we included articles in this review that assess the effect of attributions on reactions toward poor performance. The majority of the studies tested behavioral and socio-emotional group reactions as a result of different attributional processes (e.g., cause) for the poor performance. Jackson and LePine (2003) manipulated the motivation (high vs. low effort) and ability (high vs. low) of poor performers in vignettes. When the poor performance was attributed to a low ability, peers were less likely to respond negatively compared to when the poor performance was attributed to low effort: they were more willing to compensate for and train the poor performer and less likely to motivate (e.g., via threats) and reject (e.g., ostracize) the poor performer.

In a vignette study, Taggar and Neubert (2008) asked participants to evaluate a poor performer with either low or high cognitive ability on the perceived degree of free-riding, the attribution locus of causality (internal vs. external), controllability, stability, emotional responses (e.g., anger) and intentions to help and to punish. Low performers with high cognitive ability received higher free-riding ratings than low performers with low ability, which in turn were associated with an attributed internal locus of causality, low stability, and high controllability. High controllability was associated with higher anger, and anger was positively related to the intention to punish. Controllability attributions were positively associated with ability and negatively with motivation, which fits classic attribution theory.

The aforementioned M-PGI has gained supporting evidence in several experimental studies by Thürmer (2024) and Thürmer & Kunze (2023). In six vignette tasks and one simulated group task, the proposed interaction effect between performance cause (low effort vs. low ability) and assumed precursors (traditional vs. reversed/M-GPI) was consistently observed: low effort only produced more negative reactions [based on Jackson & LePine (2003) scales] than low ability when a desirability attribution was made for effort and a feasibility attribution was made for ability. The effect was not present for the reversed assumption linking effort to feasibility and ability to desirability. Additionally, the present effect was fully mediated by the performer’s perceived pro-group intent and emotional responses. The desirability/feasibility distinction has been proven to be highly relevant for the evaluation of poor performance.

Although the M-PGI has not been tested in face-to-face interaction with the poor performer, other studies have observed responses to poor performers in interacting teams. Taggar and Neubert (2004) examined, in Study 1, reactions toward a poor performing team member varying in cognitive ability (high vs. low) and conscientiousness (high vs. low), finding that causal attributions (locus, controllability, and stability), emotional responses, and behavioral intentions to help and punish were affected (sig. main effects and interaction). In Study 2, they assessed student groups over a semester and identified each group’s lowest-performing member. In line with attribution theory, the personality traits of these members predicted group responses: individuals low in conscientiousness but high in intelligence, suggesting low effort despite sufficient ability, elicited less prosocial behavior from others than those with other trait combinations.

The willingness to compensate, which has only been measured in self-report by others (e.g., Jackson & LePine, 2003), was also tested in an interactive group task. Kerr (1983) tested behavioral reactions when working with a low-performing coworker on a physical motor production task. When the poor performance of a coworker was attributed to low ability, individuals increased their performance relative to the individual condition, and they engaged in social compensation. In contrast, when the poor performer was high in ability—and therefore apparently lacking the motivation to perform well—participants reduced their effort, to avoid being exploited (*sucker effect*).

There is also contrasting evidence: Gupta (2012) examined four-person teams in which a low-performing confederate was present and either a low effort or low ability attribution was offered. Contrary to predictions and previous evidence, no effect of the attribution type on team responses, perceived valence of socioemotional interaction, or perceived team conflict and cohesion was found.

In interactive groups, reactions of group members may additionally vary depending on whether decisions to punish are taken consensually or individually. When individual versus joint responses to poor performers are compared, group interaction can alter attribution processes and subsequent reactions. Liden et al. (1999) evaluated the severity of disciplinary decisions in hypothetical scenarios presented to 41 work groups (and their managers) in different organizations. The consensual group decisions turned out to be more severe when an internal attribution was offered as a cause for a poor performance (vs. external attribution). Interacting groups made more severe disciplinary decisions compared to decisions made by group members individually.

### 6.1. Synthesis and Summary of Results

In light of our findings, instead of assuming that perspective-taking has ubiquitous positive consequences in group interaction, we find that it also enables attribution processes that elicit negative peer reactions towards poor performers. These negative reactions can have a downstream effect, impairing team efficiency and fueling social conflict. Although perspective-taking has been shown to be beneficial in various group settings, it would not suffice to instruct groups to engage in perspective-taking to tackle obstacles posed by poor performance. In contrast to the intensity of such an effort, the outcome and inferences drawn as a result of perspective-taking determine the reaction. The presented evidence stresses the importance of considering the attribution process underlying perspective-taking efforts to understand group reactions to poor performers. While it has been established that the cause of poor performance (e.g., ability vs. effort) shapes the reaction toward poor performance (Liden et al., 1999; Jackson & LePine, 2003; Kerr, 1983; Taggar & Neubert, 2004, 2008), more recent evidence stresses the necessity for nuanced predictions. The process view of the M-PGI helps understand how groups evaluate poor performers and adjust their responses accordingly. When the groups engage in perspective-taking and consider the point of view of a team member who shows a lower contribution to the collective goal, they make inferences about the target’s abilities, efforts, and intentions. In that, they not only focus on the observable characteristics (effort/ability) but also the subjective goal-setting process (desirability/feasibility) (Figure 1B). Taken together, perspective-taking should lead to highly nuanced attributions of causes for poor performance and—following the M-PGI—consequently the ascription of high or low pro-group intent. In turn, the degree of attributed pro-group intent shapes the group’s reactions.

### 6.2. Limitations

Our scoping review has some limitations. Due to the exploratory nature of the scoping review, we did not assess the quality or risk of bias of the included studies, which limits the ability to evaluate the robustness of the evidence. Additionally, our narrow inclusion criteria bear the risk of missing relevant literature. Yet, an expansion of the search criteria would have a very different focus and be less concerned with how groups respond to poor performance. The wide variety of study designs does not allow for drawing robust conclusions in the form of a quantitative synthesis; instead, the scoping review approach allowed for mapping the evidence concerning our research question and evaluating the results in a narrative synthesis. Considering the negative effects of poor performance in teams and the general focus on the beneficial effects of perspective-taking, the lack of evidence is a cause for concern. Thus, our paper is a call for renewed efforts to understand group reactions to poor performers, merging the evidence on perspective-taking and attribution of intent.

## 7. Discussion

In this review, we addressed the importance of perspective-taking in social interaction and collective action control in groups. We reviewed evidence showing that attribution of intent is a key predictor of reactions to poor performers. Thus, group reactions depend on nuanced attributions instead of perspective-taking. The Model of Pro-Group Intent (M-PGI) postulates that groups respond differently to poor performance. The attribution of pro-group intent provides a highly parsimonious account of whether a group supports and compensates for a poor performer or excludes and punishes them to protect group performance, for instance relying only on one mediator rather than the three mediators typically included in past models (Jackson & LePine, 2003; LePine & Van Dyne, 2001).

More broadly, our review contributes to the existing research on perspective-taking, reactions toward poor performers in groups, and pro-group intent. As we discussed the case of poor performers in teams in particular, it is important to stress that other forms of diverging behavior in groups exist, especially in the organizational context (Gunia & Kim, 2016). For example, dealing with and especially rejecting critical feedback (cf. Thürmer et al., 2024) can also be understood via attributional processes, emphasizing that reactions are shaped more by the perceived intent of the commenter than by the content of the critique itself. Research consistently demonstrated that perceptions of message constructiveness (Hornsey & Imani, 2004), which reflect elements of intentionality, play a key role in this dynamic. The rejection of criticism could therefore be a consequence of the attribution of a lower pro-group intent to the sender of the message.

While the consistent empirical support indicates systematic variance in understanding others’ intentions, it is important to note that attributions can be incorrect (cf. Eyal et al., 2018). Reactions toward a poor performer could be unjustified or inefficient. In particular, having a judgment that relies solely on ability and effort attributions while ignoring the genesis of these behaviors in terms of subjective desirability and feasibility may lead to overgeneralization. Specifically, desirability/feasibility assumptions were an important moderator of group reactions toward poor performers (Thürmer & Kunze, 2023). Ignoring this aspect could trigger potentially false reactions that could be considered unjust towards the performer.

Furthermore, it can be advantageous for short-term group goals to exclude a poor performer instead of dragging them along (Jackson & LePine, 2003; LePine & Van Dyne, 2001), but the ecological reality in the working world is much more complex. In addition to performance, individuals also fulfill other essential roles in group dynamics, for example motivators or leaders (cf. Grant, 2023). Grant (2023) highlighted that group success often depends on recognizing and leveraging the hidden potential of individuals, including those who may initially appear as deviant, by considering their intent and contributions to the collective. Also, individuals who behave in a prosocial manner by contributing a lot to a group goal (*prosocial behavior*) do not necessarily act out of a concern for the well-being of others (*prosocial motivation*) or have a prosocial impact with this behavior (*prosocial impact*; cf. Bolino & Grant, 2016). Here, the pro-group intent of a poor performer should determine which reactions could be profitable for a group. Sorting between good and bad apples based on pro-group intent should ensure group success and efficiency in the long term. Investigating this is a fruitful task for future research.

In this review paper, we shed light on the complex dynamics of dealing with diverging performance in groups using the example of poor performers. Groups engage in collective action to control and regulate the behavior of their members, to effectively achieve goals cooperatively. This capacity is particularly relevant when group members deviate from others in their contribution to the group goal. Group reactions toward poor performers are not solely determined by perspective-taking efforts per se but rather by the content of the attributional process. When dealing with poor performers, it is not only important to try to understand the reasons for the person’s diverging behavior by putting yourself in his or her shoes, but also what the conclusion of the efforts is. Drawing on the M-PGI, we argued that perceived pro-group intent is a significant factor in understanding responses to poor performers. Our findings highlight the importance of differentiating different types of Theory of Mind processes for understanding group functioning. Specifically, our review highlights that perspective-taking and attribution of intent are qualitatively different processes that have nuanced effects on the reaction to poor performers in teams. In applied contexts, such as organizations or social settings, group members routinely face underperformance. How they interpret the causes of such performance can critically shape their responses. Thus, integrating Theory of Mind into models of group dynamics may prove useful for understanding how groups manage goal-threatening behavior in complex real-world environments.

## Data Availability

We have uploaded the imported literature search results in OSF: https://osf.io/r3dn4/?view_only=6de5a30d6e2e4cd497e722443ee3e2bb (accessed on 17 April 2025).

## Supplementary Materials

The following supporting information can be downloaded at: https://www.mdpi.com/article/10.3390/bs15050612/s1, Table S1: Summary of included studies.
