# Supplementary material for: Perspective-Taking and Reactions Toward Poor Performers in Groups: A Scoping Review and Discussion
Source: Behav Sci (Basel). 2025 May 1;15(5):612. doi: 10.3390/bs15050612 (PMC12108877; doi:10.3390/bs15050612)
Supplement: Supplementary file 1 [file behavsci-15-00612-s001.zip › behavsci-3452958-supplementary.pdf]

Supplementary Material

Table S1. Summary of included studies.

Authors	Sample	Method	Group task	Predictors/ Intervention	Key dependent variables	Relevant key findings
Caruso et al. (2006b)	Study 3: *N* = 136	Experiment	Group essay task	2 (High vs. low contribution) × 2 (self- vs. other-focus)	- Perceived percentage of personal contribution<br>- Enjoyment with the group<br>- Desire to work with the group in the future<br>- Happiness with division of labor	- Individuals in the other-focus condition reported lower personal contributions than those in the self-focus condition (decreased egocentric bias), no sig. interaction with high vs. low contribution manipulation<br>- When considering others' contribution, high contributors enjoyed the project less and were less happy with the division of labor than low contributors
	Study 4: *N* = 70	Experiment	Remembered group task	2 (Competitive vs. cooperative group task setting) × 2 (self- vs. other-focus)	- Perceived percentage of personal contribution<br>- Enjoyment with the group<br>- Desire to work with the group in the future	- Decreased egocentric bias when estimating responsibility allocation in the other-focus condition<br>- High performers in the other-focused cooperative condition showed lower desire for future collaborations than low performers (no effects in the self-focused cooperative or the competitive conditions)
Reimer, T. (2001)	N = 80	Experiment	Tower of Hanoi problem	2 (Trained in different strategies to solve the task: recursion vs. movement) × 2 (pair composition: uniform vs. mixed)	- Percentage of correct problem solving<br>- Attribution and blame (self vs. other attribution) for poor performance<br>- Evaluation of performance	- Poor performance was primarily attributed to the partner (vs. to the self)<br>- the more poor performance was attributed to the self, the higher the group performance in the subsequent task
Jackson, C. L. & LePine, J. A. (2003)	*N* = 217	Experiment	Vignettes	2 (motivation: high vs. low) × 2 (ability: high vs. low) × 2 (compliance: high vs. low) of low performers	- peer responses (compensation, motivation, training, rejection)<br>- Attributions: Controllability and Stability<br>- Sympathy<br>- Expectancy for change	- All three independent variables were sig. associated with peer responses: when poor performer was low in ability, peers were more likely to compensate and train, and less likely to motivate and reject; when the poor performer was low in motivation, peers were more likely to motivate and reject, and less likely to compensate and train.<br>- Controllability attributions were positively associated with ability and negatively with motivation

Taggar, S. & Neubert, M. (2008)	*N* = 268	Experiment	Video vignettes	Cognitive ability (*g*) (high vs. low) of the poor performer	- perceived free-riding<br>- Attribution of locus of causality (internal vs. external), controllability, and stability<br>- Emotional responses (anger, sympathy)<br>- Expectations of future job performance<br>- Intentions (help, punish)	- Free-riding ratings are higher for high-g low performers (compared to low-g)<br>- High free-riding was associated with internal locus, low stability, and high controllability<br>- High controllability attributions were associated with higher anger, and higher anger with a higher intention to punish
Thürmer, J. L. & Kunze, F. (2023)	Study 1: *N* = 205	Experiment	Vignettes	2 (Performance Cause: low effort vs. low ability) × 2 (Assumptions: traditional vs. reversed)	- negative peer responses - Emotional responses - Perceived pro-group intent of poor performer	- Under traditional assumptions, participants reported more negative group reactions when the poor performer was low in effort (compared to low ability) - This effect is not present under reversed assumptions - participants ascribed lower pro-group intent when poor performance was due to low effort (traditional assumptions) compared to ability, but the effect was reduced under reversed assumptions - sig. indirect effect of interaction on group reactions via pro-group intent and emotional responses
	Study 2: *N* = 200	Experiment	Vignettes	- see Study 1	- see Study 1 (modified)	- see Study 1
	Study 3: *N* = 197	Experiment	Vignettes	- see Study 1	- see Study 1 (modified)	- see Study 1
	Study 4: *N* = 198	Experiment	Vignettes	- see Study 1	- see Study 1 (modified)	- see Study 1
	Study 5: *N* = 211	Experiment	Office task	- see Study 1	- Bonus payment for poor performer<br>- willingness to work with the poor performer again<br>- Emotional responses<br>- Perceived pro-group intent of poor performer	- Under traditional assumptions, participants ascribed lower bonus payments and lower willingness to work with the poor performer when poor performer was low in effort (vs. ability), but not under reversed assumptions<br>- The effect of 2 x 2 interaction on the decision to work with poor performer and bonus was mediated by pro-group intent and emotional responses

Thürmer, J. L. (2024)	Study 1: *N* = 65	Experiment	Vignettes	2 (Performance Cause: low effort vs. low ability) × 2 (Assumptions: traditional vs. reversed)	- willingness to work with the poor performer again<br>- responsibility for poor performance	- Poor performers' lack of effort (ability) was perceived as more responsible for their poor performance in the low effort (low ability) condition compared to low ability (low effort)<br>- no effect of assumptions, no interaction<br>- Under traditional assumptions, participants were more likely to exclude the target from future group work when they were low in effort than low in ability (traditional), no sig. difference under reversed assumptions
	Study 2: *N* = 98 dyads	Experiment	Vignettes	2 between (dyadic interaction: yes vs. no) × 2 within (performance cause: low effort vs. low ability) × 2 within (assumptions: traditional vs. reversed)	- willingness to work with the poor performer again<br>- Emotional responses<br>- Perceived pro-group intent of poor performer	- Under traditional assumptions, participants were more likely to exclude the target from future group work when they were low in effort than low in ability, no sig. difference under reversed assumptions<br>- Pro-group intent significantly predicted participants' emotional response toward the poor performer, and this emotional response predicted whether participants decided to work with the poor performer again<br>- Pro-group intent and emotional responses were mediators of the effect of the performance cause × assumption interaction
Taggar, S. & Neubert, M. (2004)	Study 1: *N* = 377	Experiment	Video vignettes	2 (Cognitive ability (*g*): high vs. low) × 2 (Conscientiousness: high vs. low)	- Cause of the poor performing team members' behavior: locus, controllability, and stability - Emotional responses - Expectations for future behavior - Behavioral responses (intentions to help and to punish) - Perceived individual team member performance	- *g* ($\eta2$ =.68) and Conscientiousness ($\eta2$ =.68) both sig. affects the dependent variables (composite score), significant interaction ($\eta2$ = .64), sig. mediation models
	Study 2: *N* = 480 (94 Teams)	Correlational design	13 different group tasks	Individual characteristics (*g* and Conscientiousness) of the poor performer (of each team)	- Prosocial behavior of the team members<br>- Team performance (13 trials)	- Teams with a poorly performing high g/ low Conscientiousness member yielded the lowest levels of prosociality (compared to other combinations of g and Conscientiousness)

Kerr (1983)	*N* = 75	Experiment	Physical motor-production task	individual vs. co-worker with (high ability/ succeeding) vs. (high ability/ failing) vs. (low ability/ failing) co-worker	- proportion of trials on which the participant succeeded (performance)	- Reduction of performance in the high ability /succeeding condition (free riding) and in the high ability / failing condition (sucker effect), when the failing was attributed to low ability, the performance was higher (social compensation) (vs. individual condition)
Gupta, N. (2012)	*N* = 299	Experiment	Team decision making	2 (transactive memory structure: integrated vs. differentiated) × 2 (attribution type: low ability & high effort vs. low effort & high ability)	- team responses (performing, facilitating, coercing)<br>- Valence of socioemotional interaction<br>- Perception of team cohesion and team conflict	- No effect of attribution type on team responses, perception of conflict, cohesion, or valence of socioemotional interactions
Liden et al. (1999)	*N* = 231 (41 groups)	Experiment	Scenarios applied to work groups	2 (attributional cause for poor performance: external vs. internal cause) × 2 (reward structure: individual-based vs. group-based) × 2 (outcome seriousness: mild vs. serious) × 3 (source of decision maker: manager, interacting group, individual group member) × 5 (organization: university, small manufacturing company, large manufacturing company, large distribution company)	- Severity of disciplinary decision (no punitiveness, low punitiveness, moderate punitiveness, and termination)	- Groups' disciplinary decisions were more severe under the internal attribution condition and less severe in the external attribution condition<br>-Interacting groups and managers (equally strict) made higher disciplinary decisions compared to individual group members<br>- Attributions of poor performance (internal vs. external) had a greater impact on interacting members' and managers' evaluations than on individual members' evaluations

Note. Summary of the included studies. Data extraction was performed by the first author and revised by the second author.
